# Comparative analysis of NRF2-responsive gene expression in AcPC-1 pancreatic cancer cell line

**DOI:** 10.1007/s13258-014-0253-2

**Published:** 2014-12-05

**Authors:** Yong Weon Yi, Seunghoon Oh

**Affiliations:** 1 Department of Nanobiomedical Science, Graduate School, Dankook University, 119 Dandae-ro, Dongnam-gu, Cheonan-si, Chungnam 330-714 Republic of Korea; 2 Department of Physiology, College of Medicine, Dankook University, 119 Dandae-ro, Dongnam-gu, Cheonan-si, Chungnam 330-714 Republic of Korea

**Keywords:** NRF2, tBHQ, AsPC-1, Pancreatic cancer, Oxidative stress, Drug metabolism

## Abstract

NRF2 is a nuclear transcription factor activated in response to oxidative stress and related with metabolizing of xenotoxic materials and ABC transporter mediated drug resistance. We studied the expression of mRNAs under the siRNA-mediated knockdown of NRF2 and tBHQ-treated condition in AsPC-1 metastatic pancreatic cancer cell line to understand the AsPC-1 specific role(s) of NRF2 and further to investigate the relationship between drug resistance and metastatic plasticity and mobility of AsPc1. Here we show that the genes of aldo–keto reductases, cytochrome P450 family, aldehyde dehydrogenase, thioredoxin reductase, ABC transporter and epoxide hydrolase responsible for drug metabolism or oxidative stress concisely responded to NRF2 stabilization and knockdown of *NRF2*. In addition the expression of *PIR*, a candidate of oncogene and *KISS1*, a suppressor of metastasis were affected by NRF2 stabilization and knockdown. Our result provide comprehensive understanding of NRF2 target genes of drug response, oxidative stress response and metastasis in AsPc-1 metastatic pancreatic cancer cell line.

**Electronic supplementary material:**

The online version of this article (doi:10.1007/s13258-014-0253-2) contains supplementary material, which is available to authorized users.

## Introduction

Every cell is inevitably exposed to extracellular and intracellular oxidative stress, every moment (Finkel 2011; Ma 2010). The nuclear factor erythroid 2-related factor 2 (NRF2 or NFE2L2) is a master transcription factor that activates a battery of genes which have roles in oxidative stress responses, detoxifications, and drug resistances (Bryan et al. 2013; Ma 2013; Mitsuishi et al. 2012; Niture et al. 2014). NRF2 binds to a DNA element, named antioxidant response element (ARE), in the promoter regions of its target genes to activate transcription of these genes (Nguyen et al. 2003). The target genes of NRF2 includes (a) antioxidant genes such as NAD(P)H dehydrogenase [quinone] 1 (*NQO1*), heme oxygenase (decycling) 1 (*HMOX1*), superoxide dismutase [Cu–Zn] (*SOD1*), and glutamate-cysteine ligase catalytic subunit (*GCLC*); (b) detoxification genes including glutathione S-transferase A3 (*GSTA3*) and thioredoxin reductase 1, cytoplasmic (*TXNRD1*); (c) and drug resistance genes such as ATP-binding cassette sub-family G member 2 (*ABCG2*) and ATP-binding cassette, sub-family C (CFTR/MRP), member 5 (*ABCC5*) (Malhotra et al. 2010; Nguyen et al. 2003).

Reactive oxygen species (ROS), which are produced by various exogenous or endogenous sources, are double-edge swords. Under tight cellular control, ROS act as important signaling molecules to regulate diverse cellular functions including transcriptional regulation and signal transduction (Corcoran and Cotter 2013; Finkel 2011; Jennings et al. 2013; Ma 2010; Ray et al. 2012). On the contrary uncontrolled production of ROS causes various human diseases through DNA damage and impaired cellular functions via oxidative stress (Acharya et al. 2010; Caputo et al. 2012; Kakehashi et al. 2013; Kryston et al. 2011; Saeidnia and Abdollahi 2013; Storr et al. 2013). As an ROS sensor, the level of NRF2 is tightly regulated by a set of proteins through proteasome-dependent proteloysis. The well-known negative regulator of NRF2 is the Kelch-like erythroid cell-derived protein with CNC homology-associated protein 1 (KEAP1). KEAP1 binds and destabilized NRF2 through ubiquitin-dependent proteasomal degradation under normal reducing condition (Bryan et al. 2013; Mitsuishi et al. 2012; Niture et al. 2014; Storr et al. 2013). NRF2 stability is also regulated by the CR6-interacting factor 1 (CRIF1) under both reducing and oxidative stress conditions (Kang et al. 2010) and the glycogen synthase kinase 3β (GSK3β)/β-transducin repeat-containing protein (β-TrCP) axis (Chowdhry et al. 2013; Rada et al. 2011; Rada et al. 2012). It has been reported that stability of NRF2 is also regulated by competitive protein–protein interaction to inhibit NRF2-KEAP1 binding by various proteins such as p21 (Chen et al. 2009), the Wilms tumor gene on X chromosome (WTX) (Camp et al. 2012), p62 (Komatsu et al. 2010), the partner and localizer of BRCA2 (PALB2) (Ma et al. 2012), the dipeptidyl peptidase III (DPP3) (Hast et al. 2013), and the breast cancer susceptibility gene 1 (BRCA1) (Gorrini et al. 2013).

NRF2 functions as either a protector against tumorigenesis or oncogene (DeNicola et al. 2011; Kensler and Wakabayashi 2010; Loboda et al. 2008; Muller and Hengstermann 2012). Stability and activity of NRF2 is important in human diseases, especially in cancers. While NRF2 decreases tumor susceptibility in most carcinogenesis models, constitutive activation of NRF2 may enhance tumor cell proliferation and/or confer drug resistance in lung, pancreatic as well as colorectal cancer cells (Arlt et al. 2013; Bryan et al. 2013; Duong et al. 2014b; Homma et al. 2009; Hong et al. 2010; Lister et al. 2011; Mitsuishi et al. 2012; Niture et al. 2014; Singh et al. 2008; Storr et al. 2013; Yamadori et al. 2012). Indeed, NRF2 is up-regulated in many types of tumors through somatic mutations that block KEAP1-dependent regulation of NRF2 stability (Mitsuishi et al. 2012; Niture et al. 2014; Storr et al. 2013). Targeting NRF2 either by RNA interference or by small molecules inhibited tumor growth and increased efficacy of chemotherapy (Singh et al. 2008) or EGF-driven proliferation (Yamadori et al. 2012) in non-small cell lung cancer models and reduced the proliferation and drug-resistance in human lung cancer cells (Homma et al. 2009) or human pancreatic cancer cells (Arlt et al. 2013; Duong et al. 2014b; Hong et al. 2010; Lister et al. 2011). Additionally in primary murine cell models, oncogenes including K-Ras, B-Raf, and Myc increased the transcription of *Nrf2* gene to activate antioxidant and detoxification program preferable for oncogenesis (Kang et al. 2014). Under these conditions, genetic targeting of K-Ras^G12D^-driven Nrf2 impaired in vivo tumorigenesis (Kang et al. 2014). Taken together, genome-wide analysis of NRF2-responsive genes in specific cancer types will give insights on the context-dependent roles of NRF2. In this work we delineated NRF2-responsive genes in As-PC1 pancreatic cancer cell lines established from metastatic cancer cell in ascites fluid (Chen et al. 1982).

## Materials and methods

### Cell culture and reagents

AsPC-1 cells were obtained from the Korean Cell Line Bank (Seoul, Korea) and maintained in RPMI-1640 media (HyClone, Logan, UT) supplemented with 20 % FBS (Invitrogen, Carlsbad, CA) and 100 U/ml penicillin/streptomycin (Welgene, Daegu, Korea). The cells were cultured in a humidified 5 % CO_2_ incubator at 37 °C. The cell viability and cell counting were assessed by the Luna Automated Cell Counter (Logos Biosystems, Gyunggi-do, Korea). Tert-butylhydroquinone (tBHQ) was purchased from Sigma (St. Louis, MO) and stored at −20 °C dissolved in DMSO with small aliquots.

### siRNA transfection

For *NRF2* knockdown, exponentially proliferating cells were transfected with synthesized control siRNA (5′-gacgagcggcacgugcacauu-3′) or *NRF2* specific siRNA (5′-gaguaugagcuggaaaaacuu-3′) (Hong et al. 2010), both purchased from Bioneer (Daejeon, Korea) using Lipofectamine 2000 (Invitrogen) according to the manufacturer’s protocol.

### Cell cycle analysis

Cell cycle analysis was carried out by propidium iodide staining and laser detection of FL2 signal using FACSCalibur (BD Science, Franklin Lakes, NJ), and the data were analyzed by CellQuest Pro software (BD Science). After treatment (72 h for siRNA and 16 h for tBHQ treatment respectively), cells were washed with PBS, fixed 70 % ethanol, and stained with propidium iodide solution (20 μg/ml) containing RNaseA (100 μg/ml) after removal of ethanol.

### RNA extraction

Total RNA from AsPC-1 cell lines were prepared with the RNeasy Mini kit (Qiagen, Valencia, CA) according to the manufacturer’s protocols. The purity and integrity of RNA sample was evaluated by determining the OD260/230 ratio, 28S/18S ratio, peak pattern and electrophoretic migration patterns on Agilent 2100 Bioanalyzer (Agilent Technologies, Palo Alto, CA).

### Western blot analysis

After 72 h of siRNA treatment or 16 h of tBHQ treatment, AsPC-1 cells were lysed in 10 mM Tris–HCl (pH 7.0), 100 mM NaCl, 1 % triton X-100, 1 mM DTT, 20 μg/ml aprotinin, 2.5 μg/ml leupeptin, and 0.5 mM PMSF. Lysates were resolved on 10 % sodium dodecyl sulfate–polyacrylamide by gel electrophoresis (SDS-PAGE) and transferred onto 0.45 μm pore size Polyvinylidene fluoride (PVDF) membranes (Millipore, Bedford, MA), and immunoblotted with following antibodies: Cyclin B1 antibody (CST#4135, Cell Signaling Technology, Danvers, MA), Cyclin D1 (CST #2922, Cell Signaling Technology), NRF2 (sc-103032, Santa Cruz Biotechnology, Santa Cruz, CA), Erk-1 (sc-94, Santa Cruz Biotechnology), Cyclin A (sc-239, Santa Cruz Biotechnology). Horseradish peroxidase (HRP)-conjugated goat anti-rabbit (sc-2004, Santa Cruz Biotechnology) or anti-mouse antibodies (sc-2005, Santa Cruz Biotechnology) were used as secondary antibodies.

### cDNA microarray analysis

The cDNA microarray analysis was carried out with fluorescence labeling of cRNA and hybridization using 4 × 44 K Human whole genome microarray (Agilent technologies, Palo Alto, CA) for tBHQ treated cells. For cDNA microarray analysis of NRF2 siRNA treated cell, Ilumina Biochip system (HT-12) was used. For each microarray three RNA samples of independent experiment were used.

### Statistical analysis

Data were analyzed by either Student’s *t* test (tBHQ treated sample) or LPE test (siRNA treated sample) (Jain et al. 2003) and the results have been expressed *p* values and mean values.

## Results and discussion

The AsPC-1 pancreatic cancer cell line, used in this work had been established from metastatic abdominal ascites fluid cells originated from metastatic pancreatic cancer (Chen et al. 1982). It contains well known mutations of pancreatic cancer including, KRAS (p.G12D), TP53 (p.C135fsP35), SMAD4 (p.R100T), and other mutations common in cancers as well: COL2A1 (c.915 + 3A > G), FBXW7 (p.R465C), HEY1 (p.I178V), KIF5B (p.Q467K), MLL (p.P3536H), RNF43 (p.S720*) (Deer et al. 2010). The relative expression level of NRF2 between various pancreatic cancer cell lines including immortalized human pancreatic ductal epithelial cell lines (HPDE) using GEO2R analysis with pre-deposited microarray data (Thu et al. 2014) at NCBI Gene Expression Ominbus (http://www.ncbi.nlm.nih.gov/geo/geo2r/?acc=GSE40099&platform=GPL6480) is presented in supplementary Fig. 1. NRF2 was reported to be increased in pancreatic cancer cell lines and the nuclear level of NRF2 in AsPC-1 cell line has been reported to be relatively higher than in immortalized pancreatic ductal epithelial cells (Hong et al. 2010; Lister et al. 2011).

**Fig. 1:**
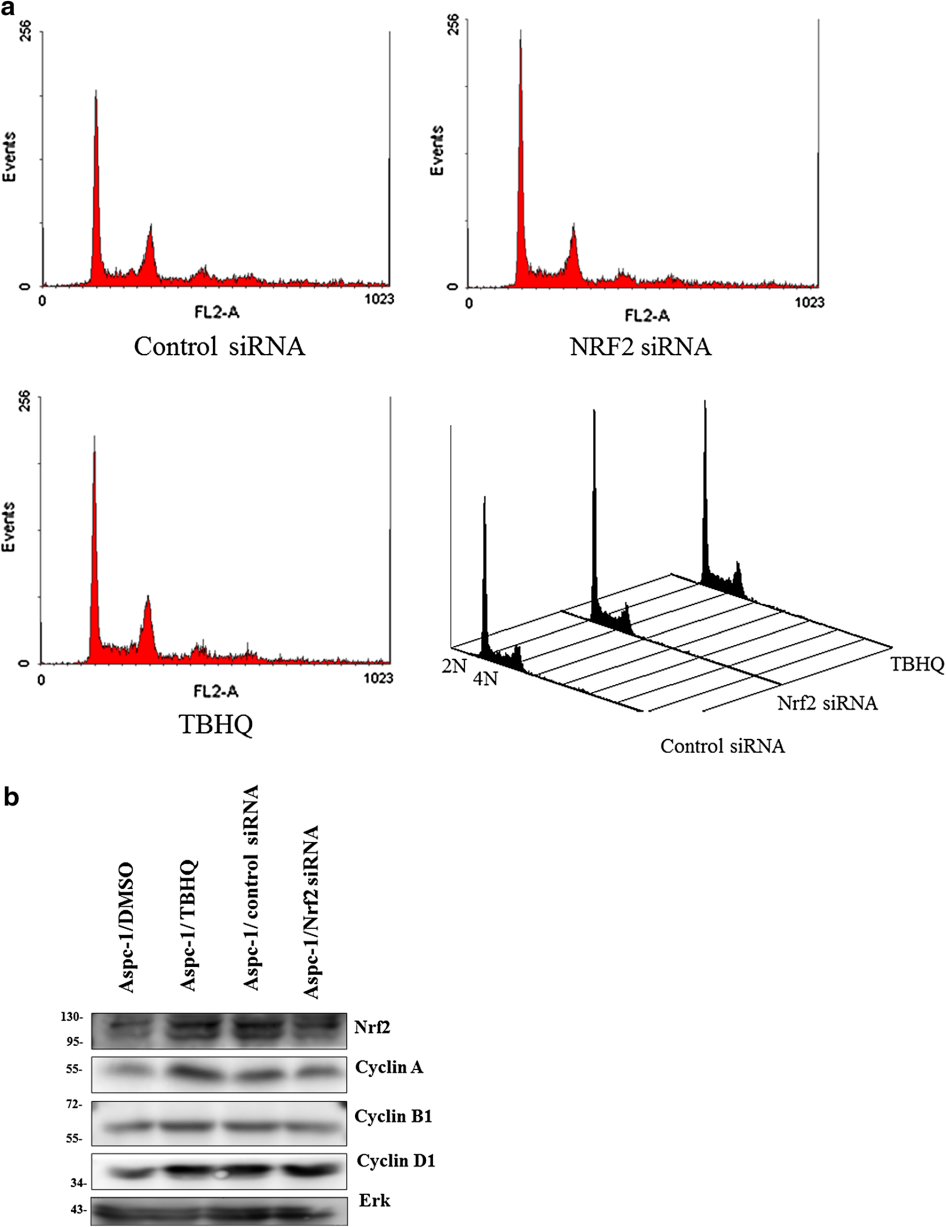
Cell cycle analysis of NRF2 siRNA-treated or tBHQ-treated AsPC-1 cell line. **a** A representative image of FL2 histogram of FACS analysis. The figure in the *lower right quadrant* is combined FACS analysis images with notion of 2 and 4 N nuclear ploidy. **b** Immunoblot anaylysis of tBHQ-treated (100 μM 16 h) or siRNA-treated samples (72 h). AsPC-1 cells were seeded in 6-well plates and treated with tBHQ (or DMSO) or NRF2 siRNA (or control siRNA). Cells were harvested and whole cell lysates were prepared, electrophoresed and transferred onto PVDF membranes. Immunoblotting was performed with indicated antibodies and Erk-1 was used as loading control

An antioxidant tBHQ increases the level of NRF2 protein by stabilization and stimulates the expression of oxidative stress metabolizing genes (Hirose et al. 1993; Li et al. 2005). Prior to cDNA microarray we tested whether tBHQ or NRF2 siRNA treatment can change the cell cycle of AsPC-1 cell line. As shown in Fig. 1a no apparent change in cell cycle distribution was observed along with no accumulation of sub G1 population. Immunoblot analysis also revealed that no apparent change of cell cycle marker proteins including cyclin B1 and cyclin D. The level of NRF2 protein was shown to be increased in tBHQ treated cells and decreased in NRF2 siRNA treated sample (Fig. 1b).

**Fig. 2:**
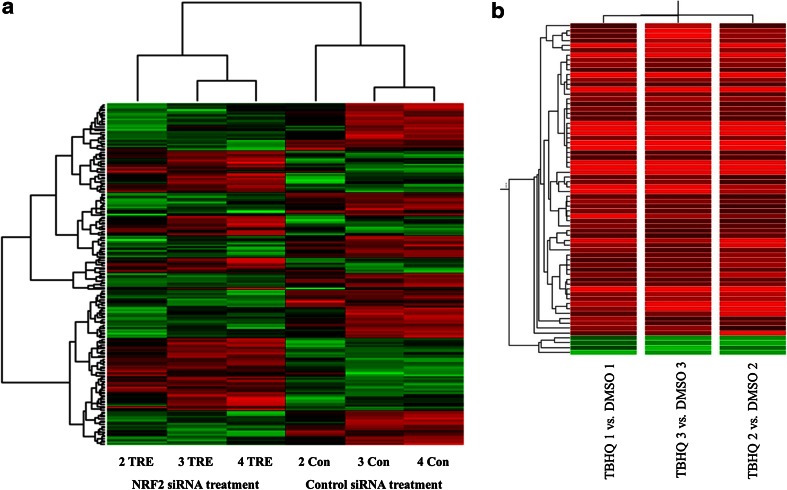
Hierarchical cluster image of the gene expression profiles of cDNA microarray analysis. **a** cDNA array of NRF2 siRNA-treated (2TRE, 3 TRE, 4 TRE) and control siRNA-treated sample (2 Con, 3 Con, 4 Con). Each mRNA sample was labeled and hybridized with cDNA array chip (HT 12, Ilumina) and cluster analysis was carried out. **b** cDNA array of tBHQ-treated versus DMSO-treated AsPC-1 cells. Two sets of mRNA (tBHQ vs DMSO) with triplicate samples were labeled differently and hybridized. The *red color* indicated up-regulated genes and the *green color* indicates down-regulated genes

To identify changed genes upon treatment of 100 μM tBHQ, we used the Agilent 44 k whole genome cDNA array chip. We also used the Ilumina HT-12 whole genome cDNA array chip for NRF2 siRNA mediated gene expression analysis. Three independent RNA samples were used in these experiments. After removal of marginal or absent signal spots, 20,312 positive spots were obtained from tBHQ-treated sample and 16,423 positive spots were obtained from NRF2 siRNA-treated sample. Hierarchical cluster image of NRF2 siRNA treatment samples reveals that the gene expression pattern of three siRNA-treated sample and three control siRNA-treated samples are adequately clustered (Fig. 2a). Figure 2b shows the hierarchical cluster image of cDNA microarray of tBHQ-treated sample indicating three independent samples share concordant RNA expression pattern.

Further statistic tests after normalization of positive spots provide statistically significant 533 array sets from tBHQ-treated samples (supplementary Table 1) and 189 array sets from NRF2 siRNA-treated samples (supplementary Table 2). Table 1 shows a list of genes which show more than two fold increase of expression (p < 0.05) after treatment of tBHQ (57 genes). Among them *AKR1B10*, *FCER1G*, *AKR1B1*, *AKR1B15*, *AADAC*, *GRK5*, *HDAC9*, *AKR1C1*, *CYP4F3*, *CYP4F2*, *ALDH3A1*, *FANCD2*, *TXNRD1* and *SLC7A11* are classified as members of drug response genes or oxidative stress response genes according to gene ontology (Table 1). The lists of genes decreased by tBHQ treatment are listed in Table 2. Four genes classified as drug response or oxidative stress response genes were identified: *PDE7A*, *TGM1*, *CYTH1* and *EPS15*. The list of top 50 genes which were decreased by NRF2 siRNA treatment are presented in Table 3. The listing is arbitrary but these genes showed more than 40 % reduction in expression. The siRNA mediated knockdown of *NRF2* significantly reduced the expression of oxidative stress or drug response genes including, *AKR1B10*, *ALDH1A1*, *HGD*, *TFF1*, *GPX2*, *ALDH3A1*, *PPP1R1B*, *AKR1C4*, *ABCB6*, *ABCC3*, *NFE2L2*, *EPHX1*, *ASGR1*, *SLC2A5*, *LGALS1* and *MTR* (Table 3). The expression of *NRF2* itself was significantly (p < 0.001, 50 % reduction) decreased by the treatment of siRNA reflecting the reliable quality control of siRNA treatment. On the contrary to NRF2 siRNA treatment the change of *NRF2* expression by the tBHQ treatment was not significant (data not shown) since tBHQ stabilized NRF2 protein but had no effect on the mRNA level of NRF2. The array results of increased genes under the NRF2 activated status (tBHQ treatment) and decreased genes by the NRF2 siRNA treatment seem to be coincide. We listed top 50 gene records with increased expression upon NRF2 siRNA treatment in Table 4. Ten genes classified as drug response or oxidative stress response genes were identified as increasing genes in NRF2 siRNA treatment: *CD36, ALPP, HLA*-*B, TGM2, FABP3, CTSH, CYR61, TIMP2, PRNP* and *NR4A2*. We also analyzed metastasis related genes in Tables 1, 2, 3 and 4.

**Table 1:** List of genes with (57 records) with more than two fold increase (p < 0.05) in tBHQ-treated AsPC-1 cells

Probe ID (Agilent 44 k)	Gene symbol	Fold change	p Value	Gene name
A_23_P415015	ATL2	10.528	5.52E−04	Atlastin GTPase 2
A_33_P3416588	RIT2	10.341	6.05E−04	Ras-like without CAAX 2
A_23_P83134	**GAS1** ^**M**^	4.752	1.13E−03	Growth arrest-specific 1
A_33_P3257155	SMAP1	4.697	4.41E−05	Small ArfGAP 1
A_24_P129341	**AKR1B10** ^**D, O**^	4.694	8.83E−04	Aldo–keto reductase family 1, member B10
A_23_P93641	**AKR1B10** ^**D, O**^	4.652	8.91E−04	Aldo–keto reductase family 1, member B10
A_33_P3272628	**FCER1G** ^**D**^	4.621	1.31E−04	Fc fragment of IgE, high affinity I, receptor for; gamma polypeptide
A_23_P258190	**AKR1B1** ^**D, O**^	4.588	9.03E−04	Aldo–keto reductase family 1, member B1
A_23_P80570	**AADAC** ^**D**^	4.488	9.31E−04	Arylacetamide deacetylase (esterase)
A_33_P3244640	**GRK5** ^**D, M**^	4.433	1.06E−03	G protein-coupled receptor kinase 5
A_33_P3380992	**AKR1B15** ^**D**^	4.415	9.39E−04	Aldo–keto reductase family 1, member B15
A_33_P3304688	TNAP	4.272	1.06E−03	TRAFs and NIK-associated protein
A_23_P404162	**HDAC9** ^**D, O, M**^	4.176	1.07E−03	Histone deacetylase 9
A_33_P3254751	LOC100131355	3.703	1.67E−03	Hypothetical protein LOC100131355
A_33_P3265394	WDR74	3.071	2.51E−03	WD repeat domain 74
A_23_P257971	**AKR1C1** ^**D, O, M**^	3.005	1.59E−03	Aldo–keto reductase family 1, member C1
A_23_P323143	ZNF767	2.919	1.76E−04	Zinc finger family member 767
A_33_P3350853	LOC202781	2.885	1.74E−04	Hypothetical LOC202781
A_23_P96623	OPN1MW	2.879	2.65E−03	Opsin 1 (cone pigments), medium-wave-sensitive
A_33_P3396956	C1orf172	2.874	1.87E−03	Chromosome 1 open reading frame 172
A_23_P67453	TNNI3	2.846	2.95E−04	Troponin I type 3 (cardiac)
A_23_P46238	CELA2A	2.823	2.08E−03	Chymotrypsin-like elastase family, member 2A
A_24_P943949	LRRC8B	2.775	3.48E−03	Leucine rich repeat containing 8 family, member B
A_23_P125042	ZNF222	2.763	3.62E−03	Zinc finger protein 222
A_33_P3268234	KRT39	2.692	3.44E−03	Keratin 39
A_32_P180741	TNK2	2.690	3.52E−03	Tyrosine kinase, non-receptor, 2
A_24_P68908	LOC344887	2.600	2.11E−03	Similar to hCG2041270
A_33_P3314401	CLDN16	2.580	3.96E−03	Claudin 16
A_33_P3365117	**AKR1C1** ^**D, O, M**^	2.563	4.45E−03	Aldo–keto reductase family 1, member C1
A_24_P152968	**AKR1C1** ^**D, O, M**^	2.562	2.18E−03	Aldo–keto reductase family 1, member C1
A_23_P63432	RHBDL2	2.509	2.89E−03	Rhomboid, veinlet-like 2 (Drosophila)
A_33_P3294277	**CYP4F3** ^**D**^	2.489	2.59E−03	Cytochrome P450, family 4, subfamily F, polypeptide 3
A_23_P28697	HAAO	2.394	4.38E−03	3-hydroxyanthranilate 3,4-dioxygenase
A_24_P678418	DICER1-AS	2.378	2.74E−03	Hypothetical locus FLJ45244
A_23_P46222	TRIM46	2.370	2.85E−03	Tripartite motif containing 46
A_33_P3389363	C19orf54	2.364	3.17E−03	Chromosome 19 open reading frame 54
A_23_P502047	CHRD	2.345	3.99E−03	Chordin
A_23_P50710	**CYP4F2** ^**D**^	2.340	4.52E−03	Cytochrome P450, family 4, subfamily F, polypeptide 2
A_33_P3315239	ZNF7	2.337	4.01E−03	Zinc finger protein 7
A_33_P3336287	SEC61A2	2.322	4.20E−03	Sec61 alpha 2 subunit (S. cerevisiae)
A_23_P301521	KIAA1462	2.275	6.97E−03	KIAA1462
A_33_P3420900	PATE2	2.272	1.54E−03	Prostate and testis expressed 2
A_23_P218793	XPNPEP3	2.187	3.38E−03	X-prolyl aminopeptidase (aminopeptidase P) 3, putative
A_33_P3265714	C2orf61	2.184	1.05E−02	Chromosome 2 open reading frame 61
A_33_P3252381	PCA3	2.167	1.36E−03	Prostate cancer antigen 3 (non-protein coding)
A_33_P3378915	ARHGEF18	2.164	3.58E−03	Rho/Rac guanine nucleotide exchange factor (GEF) 18
A_33_P3397520	KRTAP10-12	2.137	4.86E−03	Keratin associated protein 10-12
A_24_P307135	TNXB	2.111	7.60E−03	Tenascin XB
A_33_P3259548	WDR5B	2.097	4.74E−03	WD repeat domain 5B
A_23_P38190	ORMDL3	2.084	4.14E−03	ORM1-like 3 (S. cerevisiae)
A_23_P3956	C1QTNF1	2.069	3.97E−03	C1q and tumor necrosis factor related protein 1
A_33_P3238433	**ALDH3A1** ^**D, O**^	2.063	3.96E−03	Aldehyde dehydrogenase 3 family, memberA1
A_23_P345678	**FANCD2** ^**D, O, M**^	2.046	5.27E−03	Fanconi anemia, complementation group D2
A_33_P3351120	**TXNRD1** ^**D,O**^	2.042	4.10E−03	Thioredoxin reductase 1
A_33_P3258581	LOC389791	2.032	5.19E−03	Hypothetical LOC389791
A_33_P3242623	**SLC7A11** ^**D, M**^	2.011	4.31E−03	Solute carrier family 7, member 11
A_24_P223163	NAF1	2.006	4.47E−03	Nuclear assembly factor 1 homolog (S. cerevisiae)

The fold increased/decreased values are mean of three independent samples. Superscripts were assigned to drug response genes (D), oxidative stress response genes (O) and metastasis (M) related genes according to gene ontology. These gene symbols are presented in bold style

**Table 2:** Top 50 gene records with decreased expression (p < 0.05) in tBHQ-treated AsPC-1 cells

Probe ID (Agilent 44 k)	Symbol	Fold change	p Value	Gene name
A_23_P337849	CELF3	0.398	1.10E−02	CUGBP, Elav-like family member 3
A_24_P322229	RASL10B	0.466	7.41E−03	RAS-like, family 10, member B
A_33_P3213512	COQ5	0.468	1.24E−02	Coenzyme Q5 homolog, methyltransferase (S. cerevisiae)
A_23_P60627	**ALOX15B** ^**M**^	0.475	1.30E−02	Arachidonate 15-lipoxygenase, type B
A_33_P3356004	UCKL1-AS1	0.542	3.18E−02	UCKL1 antisense RNA 1 (non-protein coding)
A_33_P3247678	LOC100130876	0.550	7.40E−03	Uncharacterized LOC100130876
A_33_P3245679	LOC100129940	0.554	2.98E−02	Uncharacterized LOC100129940
A_23_P146325	ASAP1-IT1	0.566	9.59E−03	ASAP1 intronic Transcript 1 (non-protein coding)
A_32_P110016	LOC727869	0.567	3.94E−02	Uncharacterized LOC727869
A_23_P59988	SLC35G5	0.567	2.72E−02	Solute carrier family 35, member G5
A_33_P3281363	TRIP12	0.573	1.24E−02	Thyroid hormone receptor interactor 12
A_23_P114445	MAGEE1	0.577	1.23E−02	Melanoma antigen family E, 1
A_24_P360529	**PDE7A** ^**D**^	0.589	3.23E−02	Phosphodiesterase 7A
A_23_P18055	C3orf51	0.597	1.85E−02	Chromosome 3 open reading frame 51
A_33_P3544880	LOC142937	0.622	1.05E−02	Uncharacterized protein BC008131
A_33_P3576797	LOC158863	0.622	1.17E−02	Uncharacterized LOC158863
A_24_P314597	KIAA0319L	0.631	1.73E−02	KIAA0319-like
A_33_P3272399	LOC645427	0.632	2.14E−02	Uncharacterized LOC645427
A_33_P3256500	ATXN2	0.636	2.07E−02	Ataxin 2
A_33_P3248265	LTB	0.647	2.48E−02	Lymphotoxin beta (TNF superfamily, member 3)
A_33_P3522511	KIAA0485	0.649	3.41E−02	Uncharacterized LOC57235
A_33_P3319134	LOC100506191	0.649	3.60E−02	Uncharacterized protein LOC100506191
A_24_P693321	LOC100190986	0.649	6.81E−03	Uncharacterized LOC100190986
A_23_P65618	**TGM1** ^**D**^	0.653	2.67E−02	Transglutaminase 1
A_33_P3249259	TGM6	0.656	1.57E−02	Transglutaminase 6
A_23_P108932	RPL23AP32	0.658	4.13E−02	Ribosomal protein L23a pseudogene 32
A_33_P3333777	LOC100129387	0.661	3.27E−02	Uncharacterized LOC100129387
A_23_P326142	C7orf54	0.663	3.05E−02	Chromosome 7 open reading frame 54
A_33_P3335840	WDR33	0.666	3.53E−02	WD repeat domain 33
A_33_P3324137	PRO0628	0.668	1.90E−02	Uncharacterized LOC29053
A_33_P3393010	PKDCC	0.669	2.08E−02	Protein kinase domain containing, cytoplasmic homolog (mouse)
A_33_P3321372	CNTNAP3	0.673	1.20E−02	Contactin associated protein-like 3
A_33_P3250018	HCFC2	0.673	4.56E−02	Host cell factor C2
A_33_P3762913	LOC100216546	0.677	3.29E−02	uncharacterized LOC100216546
A_33_P3223990	TPM3	0.680	3.66E−02	Tropomyosin 3
A_33_P3503937	LOC284581	0.683	1.31E−02	Uncharacterized LOC284581
A_33_P3357382	POGZ	0.685	1.94E−02	Pogo transposable element with ZNF domain
A_33_P3276913	TTC3	0.685	3.02E−02	Tetratricopeptide repeat domain 3
A_33_P3363091	VAC14	0.685	2.87E−02	Vac14 homolog (S. cerevisiae)
A_33_P3356525	FLJ45482	0.686	1.22E−02	Uncharacterized LOC645566
A_33_P3310751	LOC100132249	0.690	4.21E−02	Uncharacterized LOC100132249
A_33_P3345743	PFN1P2	0.691	2.36E−02	Profilin 1 pseudogene 2
A_23_P6561	EBLN2	0.692	1.29E−02	Endogenous Bornavirus-like nucleoprotein 2
A_23_P59613	**FZD9** ^**M**^	0.692	1.63E−02	Frizzled family receptor 9
A_33_P3397795	C14orf135	0.694	1.31E−02	Chromosome 14 open reading frame 135
A_33_P3304533	RNF207	0.696	2.21E−02	Ring finger protein 207
A_33_P3380405	**CYTH1** ^**D**^	0.699	1.98E−02	Cytohesin 1
A_33_P3538279	PRO2852	0.699	2.61E−02	Uncharacterized protein PRO2852
A_23_P60793	ASMTL-AS1	0.703	3.95E−02	ASMTL antisense RNA 1 (non-protein coding)
A_33_P3371752	**EPS15** ^**D, M**^	0.704	1.52E−02	Epidermal growth factor receptor pathway substrate 15
A_33_P3355371	TTC9C	0.704	3.17E−02	Tetratricopeptide repeat domain 9C

The fold increased/decreased values are mean of three independent samples. Superscripts were assigned to drug response genes (D), oxidative stress response genes (O) and metastasis (M) related genes according to gene ontology. These gene symbols are presented in bold style

**Table 3:** Top 50 gene records with decreased expression (p < 0.05) in NRF2 siRNA-treated AsPC-1 cells

Probe IDIlumina	Symbol	Fold change	p Value (LPE t-test)	Gene name
ILMN_1672148	**AKR1B10** ^**D, O**^	0.241	0.00E+00	Aldo–keto reductase family 1, member B10 (aldose reductase)*
ILMN_1709348	**ALDH1A1** ^**D, O, M**^	0.253	0.00E+00	Aldehyde dehydrogenase 1 family, member A1
ILMN_2096372	**ALDH1A1** ^**D, O, M**^	0.358	4.86E−12	Aldehyde dehydrogenase 1 family, member A1
ILMN_2198239	**HGD** ^**O**^	0.393	5.28E−08	Homogentisate 1,2-dioxygenase (homogentisate oxidase)
ILMN_1794829	C6orf117	0.410	1.82E−07	Chromosome 6 open reading frame 117
ILMN_1729117	COL5A2	0.418	1.54E−08	Collagen, type V, alpha 2
ILMN_1811387	**TFF3** ^**M**^	0.426	0.00E+00	Trefoil factor 3 (intestinal)
ILMN_1781745	C9orf152	0.445	1.43E−06	Chromosome 9 open reading frame 152
ILMN_1722489	**TFF1** ^**D, O, M**^	0.445	1.24E−10	Trefoil factor 1
ILMN_1800091	RARRES1	0.465	1.40E−06	Retinoic acid receptor responder (tazarotene induced) 1
ILMN_2133205	**GPX2** ^**D, O**^	0.469	3.59E−10	Glutathione peroxidase 2 (gastrointestinal)
ILMN_1702503	**ALDH3A1** ^**D, O**^	0.481	3.84E−06	Aldehyde dehydrogenase 3 family, memberA1*
ILMN_2412336	AKR1C2	0.488	3.94E−05	Aldo–keto reductase family 1, member C2
ILMN_2304495	**PPP1R1B** ^**D, O**^	0.489	1.57E−05	Protein phosphatase 1, regulatory (inhibitor) subunit 1B
ILMN_1684873	ARSD	0.491	5.26E−05	Arylsulfatase D
ILMN_1772951	ST6GALNAC1	0.492	1.06E−07	ST6 (α-N-acetyl-neuraminyl-2,3-β-galactosyl-1, 3)-N-acetylgalactosaminide α-2,6-sialyltransferase 1
ILMN_1687757	**AKR1C4** ^**O**^	0.506	1.81E−04	Aldo–keto reductase family 1, member C4
ILMN_2193980	**ABCB6** ^**D**^	0.509	5.44E−06	ATP-binding cassette, sub-family B (MDR/TAP), member 6
ILMN_2161330	**SPDEF** ^**M**^	0.513	2.61E−03	SAM pointed domain containing ets transcription factor
ILMN_1677814	**ABCC3** ^**D, O**^	0.518	3.96E−06	ATP-binding cassette, sub-family C (CFTR/MRP), member 3
ILMN_1790909	**NFE2L2** ^**D, O**^	0.519	6.27E−04	Nuclear factor (erythroid-derived 2)-like 2
ILMN_1680652	SELENBP1	0.520	3.74E−04	Selenium binding protein 1
ILMN_1756685	DEPDC6	0.523	6.27E−04	DEP domain containing 6
ILMN_1704353	IGSF3	0.525	6.27E−04	Immunoglobulin superfamily, member 3
ILMN_1743620	RARRES1	0.528	1.47E−03	Retinoic acid receptor responder (tazarotene induced) 1
ILMN_1752932	MPZL2	0.532	2.94E−03	Myelin protein zero-like 2
ILMN_1701025	**EPHX1** ^**D, O**^	0.535	4.13E−05	Epoxide hydrolase 1, microsomal (xenobiotic)
ILMN_1680738	C5orf13	0.543	6.93E−03	Chromosome 5 open reading frame 13
ILMN_1653956	LOC644624	0.545	6.70E−03	PREDICTED: hypothetical LOC6446241
ILMN_1769013	**ASGR1** ^**D, O**^	0.545	2.13E−04	Asialoglycoprotein receptor 1
ILMN_1748352	**CTSL2** ^**M**^	0.547	3.16E−03	Cathepsin L2
ILMN_1659984	MEP1A	0.550	3.94E−05	Meprin A, alpha (PABA peptide hydrolase)
ILMN_1736042	ME1	0.551	2.91E−03	Malic enzyme 1, NADP(+)-dependent, cytosolic
ILMN_1779015	ZNF467	0.554	1.05E−03	Zinc finger protein 467
ILMN_1761247	**PIR** ^**M**^	0.561	1.83E−02	Pirin (iron-binding nuclear protein)
ILMN_2255579	RAB37	0.565	6.27E−04	RAB37, member RAS oncogene family
ILMN_1726114	SLC45A3	0.566	1.96E−06	Solute carrier family 45, member 3
ILMN_1671337	**SLC2A5** ^**D, O**^	0.566	6.19E−03	Solute carrier family 2 (facilitated glc/fruc transporter), member 5
ILMN_2278335	LOC441282	0.567	4.28E−04	Similar to aldo–keto reductase family 1, member B10
ILMN_1712305	CYBRD1	0.572	6.27E−04	Cytochrome b reductase 1
ILMN_2383383	**PIR** ^**M**^	0.576	1.74E−02	Pirin (iron-binding nuclear protein)
ILMN_1657547	CCDC34	0.578	2.33E−04	Coiled-coil domain containing 34
ILMN_1678692	MPRIP	0.579	9.67E−03	Myosin phosphatase Rho interacting protein
ILMN_1723978	**LGALS1** ^**D, O, M**^	0.579	5.76E−03	Lectin, galactoside-binding, soluble, 1
ILMN_2087692	CYBRD1	0.581	1.45E−03	Cytochrome b reductase 1
ILMN_1802100	ADAM28	0.587	3.29E−02	ADAM metallopeptidase domain 28
ILMN_1761733	HLA-DMB	0.587	7.93E−03	Major histocompatibility complex, class II, DM beta
ILMN_1695397	LOC644151	0.588	1.64E−03	PREDICTED: similar to calpain 8 (LOC644151)
ILMN_1670801	**MTR** ^**D, O**^	0.591	3.80E−02	5-methyltetrahydrofolate-homocysteine methyltransferase
ILMN_1699728	BTD	0.591	1.74E−02	Homo sapiens biotinidase

The fold changes are mean of three independent samples. Superscripts were assigned to drug response genes (D), oxidative stress response genes (O) and metastasis (M) related genes according to gene ontology. These gene symbols are presented in bold style

**Table 4:** Top 50 gene records with increased expression (p < 0.05) in NRF2 siRNA-treated AsPC-1 cells

Probe ID Ilumina	Symbol	Fold change	p Value (LPE t-test)	Gene name
ILMN_1796094	**CD36** ^**D, O, M**^	4.476	4.78E−25	CD36 molecule (thrombospondin receptor)
ILMN_1784863	**CD36** ^**D, O, M**^	3.416	4.13E−13	CD36 molecule (thrombospondin receptor)
ILMN_1656501	DUSP5	2.664	1.24E−08	Dual specificity phosphatase 5
ILMN_1679262	**DPYSL3** ^**M**^	2.389	7.67E−11	Dihydropyrimidinase-like 3
ILMN_1693789	**ALPP** ^**D, O**^	2.296	1.82E−07	Alkaline phosphatase, placental (Regan isozyme)
ILMN_1700144	ITGA10	2.241	7.76E−06	Integrin, alpha 10
ILMN_1787691	CITED4	2.157	5.80E−06	Cbp/p300-interacting transactivator, with Glu/Asp-rich carboxy-terminal domain, 4
ILMN_2108735	EEF1A2	2.094	6.43E−03	Eukaryotic translation elongation factor 1 alpha 2
ILMN_1813386	CORO6	2.073	4.73E−05	Coronin 6
ILMN_2368530	**IL32** ^**M**^	2.042	1.06E−07	Interleukin 32
ILMN_1776861	HAP1	2.039	2.25E−04	Huntingtin-associated protein 1
ILMN_2317581	SHANK3	2.023	2.48E−05	SH3 and multiple ankyrin repeat domains 3
ILMN_2317580	SHANK3	1.950	1.28E−03	SH3 and multiple ankyrin repeat domains 3
ILMN_2049417	TMEM86B	1.920	8.22E−04	Transmembrane protein 86B
ILMN_1778010	**IL32** ^**M**^	1.919	2.26E−04	Interleukin 32
ILMN_1697460	REEP6	1.915	4.15E−03	Receptor accessory protein 6
ILMN_1710553	TMEM151A	1.900	2.61E−03	Transmembrane protein 151A
ILMN_1678086	CCDC74A	1.894	2.68E−03	Coiled-coil domain containing 74A
ILMN_1778401	**HLA-B** ^**D, O, M**^	1.878	8.82E−05	Major histocompatibility complex, class I, B
ILMN_1709659	TMEM151A	1.868	1.74E−02	Transmembrane protein 151A
ILMN_1734707	CHST13	1.857	3.42E−03	Carbohydrate (chondroitin 4) sulfotransferase 13
ILMN_1794501	**HAS3** ^**M**^	1.840	1.28E−03	Hyaluronan synthase 3
ILMN_1674580	TRIM36	1.834	1.67E−03	Tripartite motif-containing 36
ILMN_1761912	MGAT1	1.824	1.29E−02	Mannosyl (alpha-1,3-)-glycoprotein beta-1,2-N-acetylglucosaminyl transferase
ILMN_1679267	**TGM2** ^**D, O, M**^	1.818	1.28E−03	Transglutaminase 2
ILMN_2136971	**FABP3** ^**D, O, M**^	1.815	5.45E−03	Fatty acid binding protein 3, muscle and heart
ILMN_2077680	CLDND2	1.814	2.15E−03	Claudin domain containing 2
ILMN_1669362	IGFBP6	1.811	3.15E−06	Insulin-like growth factor binding protein 6
ILMN_2361737	TRIM36	1.809	2.94E−03	Tripartite motif-containing 36
ILMN_1805842	FHL1	1.796	1.82E−03	Four and a half LIM domains 1
ILMN_2390853	**CTSH** ^**D, O**^	1.780	2.68E−03	Cathepsin H
ILMN_1676712	LOC645553	1.778	1.28E−03	PREDICTED: hypothetical LOC645553
ILMN_2171384	**CXCL5** ^**M**^	1.766	1.17E−02	Chemokine (C-X-C motif) ligand 5
ILMN_1780057	RENBP	1.764	6.43E−03	Renin binding protein
ILMN_2188264	**CYR61** ^**O, M**^	1.759	5.85E−03	Cysteine-rich, angiogenic inducer, 61
ILMN_1782305	**NR4A2** ^**O, M**^	1.744	9.73E−03	Nuclear receptor subfamily 4, group A, member 2
ILMN_1792538	CD7	1.740	3.63E−02	CD7 molecule
ILMN_1705814	KRT80	1.738	9.51E−03	Keratin 80
ILMN_1721876	**TIMP2** ^**O, M**^	1.733	3.53E−02	TIMP metallopeptidase inhibitor 2
ILMN_1655915	**MMP11** ^**M**^	1.725	2.02E−02	Matrix metallopeptidase 11 (stromelysin 3)
ILMN_1656361	LOC201175	1.722	1.43E−02	Hypothetical protein LOC201175
ILMN_1785646	PMP22	1.720	4.71E−02	Peripheral myelin protein 22
ILMN_1748844	CNKSR3	1.713	1.29E−02	CNKSR family member 3
ILMN_2360415	**PRNP** ^**O**^	1.713	2.15E−02	Prion protein (PRNP)2
ILMN_1814296	TRPM6	1.711	2.15E−03	Transient receptor potential cation channel, subfamily M, member 6
ILMN_1667295	VASN	1.706	1.84E−02	Vasorin
ILMN_1727466	KCNMB4	1.700	5.76E−03	Potassium large conductance calcium-activated channel, subfamily M, beta member 4
ILMN_2405009	NBL1	1.695	2.38E−02	Neuroblastoma, suppression of tumorigenicity 1
ILMN_1801246	IFITM1	1.694	6.27E−04	Interferon induced transmembrane protein 1 (9–27)
ILMN_2339955	**NR4A2** ^**O, M**^	1.688	3.79E−02	Nuclear receptor subfamily 4, group A, member 2

The fold changes are mean of three independent samples. Superscripts were assigned to drug response genes (D), oxidative stress response genes (O) and metastasis (M) related genes according to gene ontology. These gene symbols are presented in bold style

In the cDNA array data of tBHQ-treated cells and NRF2 siRNA-treated samples, total 18 overlapping genes could be obtained with statistical significance (p < 0.05) (Table 5). Unexceptionally 17 genes with increased mRNA expression under the tBHQ treatment showed decreased expression by NRF2 siRNA treatment. The metastasis genes whose relationship with NRF2 was reported previously are as follows: *AKR1B10* (Agyeman et al. 2012; Nishinaka et al. 2011), *ALDH3A1* (Agyeman et al. 2012), *TXNRD1* (Sakurai et al. 2005), *AKR1C4* (Ebert et al. 2011), *ALDH1A1* (Duong et al. 2014a), *PIR* (Hubner et al. 2009), *GPX2* (Banning et al. 2005), *UGDH* (Loignon et al. 2009), *SRXN1* (Soriano et al. 2008), *ME1* (Thimmulappa et al. 2002), *ABCB6* (Campbell et al. 2013), *EPHX1* (Su et al. 2014), *NQO1* (Agyeman et al. 2012; Loignon et al. 2009; Thimmulappa et al. 2002), and *ABCC3* (Adachi et al. 2007). Interestingly, we identified three new genes including *ALDH3A2*, *ASPH*, and *KISS1* as NRF2-responsive genes in this study. To date no study has been reported the relationship of NRF2 with ALDH3A2, ASPH, and KISS1. KISS1 is a protein with 145 amino acid residues and its role is known as an inhibitor of metastasis (Ji et al. 2013). Overexpression KISS1 inhibits metastatic colony formation in ovarian cancer cell lines (Jiang et al. 2005). However, the role of KISS1 in pancreatic cancers has not yet been elucidated. Previously, a report displayed that *NRF2* deficient mice showed higher number of pulmonary metastasis than wild-type mice (Satoh et al. 2010). ShRNA mediated knockdown of *NRF2* also enhanced cellular plasticity and motility in HepG2 cell (Rachakonda et al. 2010). However, in esophageal squamous cancer cell line NRF2 suppression downregulated the migration and invasion (Shen et al. 2014). Currently, the potential role of NRF2 in regulation of metastasis is under active investigation.

**Table 5:** List of statistically significant overlapping genes between two microarray data (tHBQ mediated activation of NRF2 and siRNA mediated depletion of NRF2)

Symbol	Probe ID agilent	Probe ID Ilumina (ILMN_)	Fold change (TBHQ)	p Value	Fold change (SiRNA)	p Value (LPE t test)	Gene name
**AKR1B10** ^**D, O**^	A_24_P129341	1672148	4.694	8.83E−04	0.241	0.00E+00	Aldo–keto reductase family 1, member B10 (aldose reductase)
**ALDH3A1** ^**D, O**^	A_33_P3238433	1702503	2.063	3.96E−03	0.481	3.84E−06	Aldehyde dehydrogenase 3 family, member A1
**TXNRD1** ^**D, O**^	A_33_P3351120	1717056	2.042	4.10E−03	0.631	2.46E−03	Thioredoxin reductase 1
**PIR** ^**M**^	A_23_P137035	1761247	1.982	4.53E−03	0.561	1.83E−02	Pirin (iron-binding nuclear protein)
**GPX2** ^**D, O**^	A_23_P3038	2133205	1.971	4.64E−03	0.469	3.59E−10	Glutathione peroxidase 2 (gastrointestinal)
**AKR1C4** ^**O**^	A_33_P3272291	1687757	1.900	5.30E−03	0.506	1.81E−04	Aldo–keto reductase family 1, member C4 (chlordecone reductase; 3-alpha hydroxysteroid dehydrogenase, type I; dihydrodiol dehydrogenase 4)
**UGDH** ^**D, M**^	A_33_P3396607	1729563	1.856	5.80E−03	0.619	4.86E−02	UDP-glucose 6-dehydrogenase
**ALDH1A1** ^**D, O, M**^	A_23_P83098	1709348	1.826	6.21E−03	0.253	0.00E+00	Aldehyde dehydrogenase 1 family, member A1
**SRXN1** ^**O**^	A_23_P320113	1804822	1.779	6.92E−03	0.689	4.00E−02	Sulfiredoxin 1
ME1	A_23_P8196	1736042	1.771	7.33E−03	0.551	2.91E−03	Malic enzyme 1, NADP(+)-dependent, cytosolic
**ABCB6** ^**D**^	A_23_P5441	2193980	1.575	1.27E−02	0.509	5.44E−06	ATP-binding cassette, sub-family B (MDR/TAP), member 6
**EPHX1** ^**D, O**^	A_23_P34537	1701025	1.538	1.46E−02	0.535	4.13E−05	Epoxide hydrolase 1, microsomal (xenobiotic)
**HGD** ^**O**^	A_23_P250164	2198239	1.518	1.58E−02	0.393	5.28E−08	Homogentisate 1,2-dioxygenase
**NQO1** ^**D, O, M**^	A_23_P206661	1720282	1.496	1.72E−02	0.659	1.65E−02	NAD(P)H dehydrogenase, quinone 1
**ALDH3A2** ^**D, O**^	A_33_P3336617	1794825	1.463	1.99E−02	0.618	1.60E−02	Aldehyde dehydrogenase 3 family, member A2
ASPH	A_24_P295245	2352934	1.375	3.11E−02	0.615	2.20E−02	Aspartate beta-hydroxylase
**ABCC3** ^**D, O**^	A_23_P207507	1677814	1.330	4.09E−02	0.518	3.96E−06	ATP-binding cassette, sub-family C (CFTR/MRP), member 3
**KISS1** ^**M**^	A_23_P124892	1669404	0.771	4.70E−02	1.534	3.29E−02	KiSS-1 metastasis-suppressor

The fold change values are mean of three independent samples. Superscripts were assigned to drug response genes (D), oxidative stress response genes (O) and metastasis (M) related genes according to gene ontology. These gene symbols are presented in bold style

## Electronic supplementary material

Below is the link to the electronic supplementary material.

Supplementary material 1 (DOC 31 kb)

Supplementary material 2 (TIFF 639 kb)

Supplementary material 3 (XLS 1103 kb)

Supplementary material 4 (XLS 686 kb)
