# Supplementary material for: Comparative analysis of NRF2-responsive gene expression in AcPC-1 pancreatic cancer cell line
Source: Genes Genomics. 2014 Dec 5;37(1):97–109. doi: 10.1007/s13258-014-0253-2 (PMC4269820; doi:10.1007/s13258-014-0253-2)
Supplement: Supplementary file 1 — Supplementary material 1 (DOC 31 kb) [file 13258_2014_253_MOESM1_ESM.doc]

**Supplementary data legend**

**Supplementary table 1**

Excel table of normalized and statistically significant (p<0.05) array signal profiles obtained from TBHQ treated AsPc-1 pancreatic cancer cell line mRNA.

**Supplementary table 2**

Excel table of normalized and statistically significant (p<0.05) array signal profiles obtained from NRF2 siRNA treated AsPc-1 pancreatic cancer cell line mRNA.

**Supplementary Fig. 1**

NRF2 expression level comparison obtained from GEO2R analysis using pre-deposited cDNA microarray.

Each GSM number represents cDNA microarray sets using Agilent 4x44k human cDNA array chip (Thu et al. 2014). GSM984880 AsPC-1 cell line; GSM984881 BxPC-3 cell line; GSM984882 Capan-1 cell line; GSM984883 Capan-2 cell line; GSM984884 CFPAC-1 cell line; GSM984885 HPAC cell line; GSM984886 HPAF-II cell line; GSM984887 Hs766T cell line; GSM984888 MIAPaCa-2 cell line; GSM984889 Panc02.03 cell line; GSM984890 Panc02.13 cell line; GSM984891 Panc03.27 cell line; GSM984892 Panc04.03 cell line; GSM984893 Panc05.04 cell line; GSM984894 Panc08.13 cell line; GSM984895 PANC-1 cell line; GSM984896 Panc10.05 cell line; GSM984897 PL45 cell line; GSM984898 SU8686 cell line; GSM984899 SW1990 cell line; GSM984900 HPDE cell line GSM984880;

**Reference**

Thu KL et al. (2014) SOX15 is a candidate tumor suppressor in pancreatic cancer with a potential role in Wnt/beta-catenin signaling. Oncogene 33:279-288
