# Supplementary figures and images for: Comparative analysis of NRF2-responsive gene expression in AcPC-1 pancreatic cancer cell line
Source: Genes Genomics. 2014 Dec 5;37(1):97–109. doi: 10.1007/s13258-014-0253-2 (PMC4269820; doi:10.1007/s13258-014-0253-2)

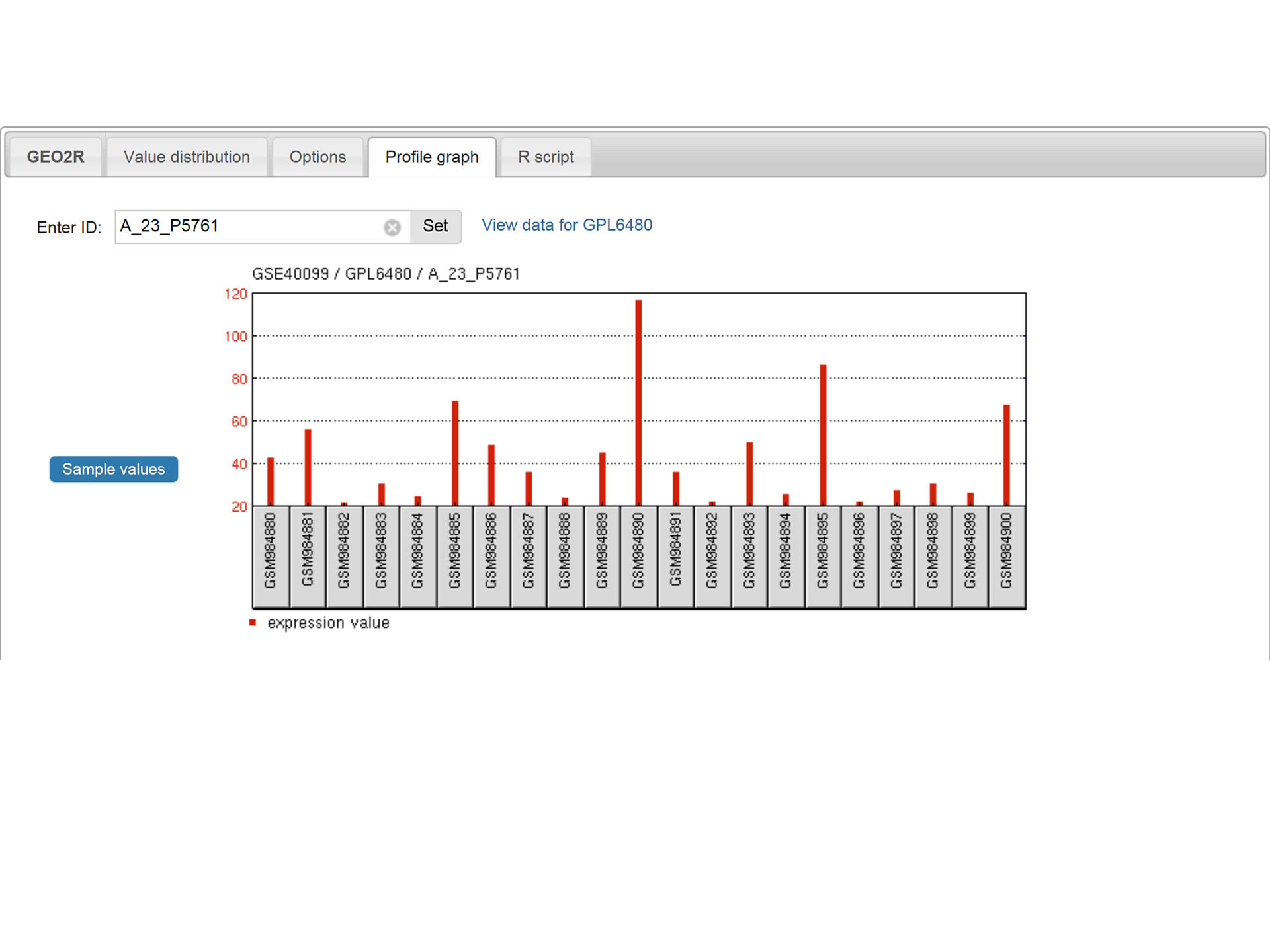

Supplement: Supplementary file 2 — Supplementary material 2 (TIFF 639 kb) [file 13258_2014_253_MOESM2_ESM.tif]
